# Angiographic Lesion Complexity Score and In-Hospital Outcomes after Percutaneous Coronary Intervention

**DOI:** 10.1371/journal.pone.0127217

**Published:** 2015-06-29

**Authors:** Ayaka Endo, Akio Kawamura, Hiroaki Miyata, Shigetaka Noma, Masahiro Suzuki, Takashi Koyama, Shiro Ishikawa, Susumu Nakagawa, Shunsuke Takagi, Yohei Numasawa, Keiichi Fukuda, Shun Kohsaka, JCD-KICS Investigators

**Affiliations:** 1 Department of Cardiology, Saiseikai Central Hospital, Tokyo, Japan; 2 Department of Cardiology, Keio University School of Medicine, Tokyo, Japan; 3 University of Tokyo, Healthcare Quality Assessment, Tokyo, Japan; 4 Department of Cardiology, Saiseikai Utsunomiya Hospital, Tochigi, Japan; 5 Department of Cardiology, National Hospital Organization, Saitama National Hospital, Saitama, Japan; 6 Department of Cardiology, Kyosai Tachikawa Hospital, Tokyo, Japan; 7 Department of Cardiology, Saitama City Hospital, Saitama, Japan; 8 Department of Cardiology, Hiratsuka City Hospital, Kanagawa, Japan; 9 Department of Cardiology, Ashikaga Red Cross Hospital, Tochigi, Japan; Niigata University Graduate School of Medical and Dental Sciences, JAPAN

## Abstract

**Objective:**

We devised a percutaneous coronary intervention (PCI) scoring system based on angiographic lesion complexity and assessed its association with in-hospital complications.

**Background:**

Although PCI is finding increasing application in patients with coronary artery disease, lesion complexity can lead to in-hospital complications.

**Methods:**

Data from 3692 PCI patients were scored based on lesion complexity, defined by bifurcation, chronic total occlusion, type C, and left main lesion, along with acute thrombus in the presence of ST-segment elevation myocardial infarction (1 point assigned for each variable).

**Results:**

The patients’ mean age was 67.5 +/- 10.8 years; 79.8% were male. About half of the patients (50.3%) presented with an acute coronary syndrome, and 2218 (60.1%) underwent PCI for at least one complex lesion. The patients in the higher-risk score groups were older (p < 0.001) and had present or previous heart failure (p = 0.02 and p = 0.01, respectively). Higher-risk score groups had significantly higher in-hospital event rates for death, heart failure, and cardiogenic shock (from 0 to 4 risk score; 1.7%, 4.5%, 6.3%, 7.1%, 40%, p < 0.001); bleeding with a hemoglobin decrease of >3.0 g/dL (3.1%, 11.0%, 13.1%, 10.3%, 28.6%, p < 0.001); and postoperative myocardial infarction (1.5%, 3.1%, 3.8%, 3.8%, 10%, p = 0.004), respectively. The association with adverse outcomes persisted after adjustment for known clinical predictors (odds ratio 1.72, p < 0.001).

**Conclusion:**

The complexity score was cumulatively associated with in-hospital mortality and complication rate and could be used for event prediction in PCI patients.

## Introduction

Percutaneous coronary intervention (PCI) is a reliable and effective therapeutic option for patients with coronary artery disease (CAD) and has become one of the most widely applied treatments in present-day cardiology. However, although periprocedural complications have declined over time, the risk of complications for patients with complex lesions in coronary vessels remains high. The definition of complex lesions includes vessel bifurcation, the presence of thrombus, involvement of the left main trunk, and the increasing number of “difficult” lesions that are now treated (e.g., those that are heavily calcified or diffuse, lesions in vessels with excessive tortuosity, or chronic total occlusive lesions) [1–9]. Therefore, it is important to evaluate the risk of complications in patients undergoing PCI for complex lesions.

No simple and user-friendly risk scoring system based on angiographic information has yet been established. The SYNTAX Score was devised to evaluate the angiographic characteristics that make a lesion suitable for either PCI or coronary artery bypass grafting (CABG), but it is not exactly a comprehensive bedside risk prediction tool [9–10]. Clinicians need a risk scoring system that will help predict short-term (i.e., in-hospital) outcomes, allowing informed clinical decisions to be made. The identification and quantification of the clinical factors associated with the complication risk would also facilitate observational research into the comparative effectiveness of therapeutic approaches. Further, at the policy-making level, predicted risk estimates can help “level the playing field” of provider outcome metrics, helping to adjust for potential differences in cases treated.

Based on the above considerations, we devised a modern PCI scoring system based on simple criteria of angiographic lesion complexity. Utilizing data from a multicenter Japanese registry, we assessed its association with in-hospital mortality and complications, as a means of facilitating more precise risk prediction.

## Methods

### Study design

The Japan Cardiovascular Database (JCD) is a large, ongoing, prospective multicenter cohort study designed to collect clinical background and outcome data on PCI patients. Data consisting of approximately 200 variables were collected for each patient. Participating hospitals were instructed to record data from consecutive hospital visits for PCI and to record them in an Internet-based database system. This system performs checks to ensure that the reported data are complete and internally consistent. PCI with any commercially available coronary device could be included. The decision to perform PCI was made according to the investigators’ clinical assessment of their patients. The study did not mandate specific interventional or surgical techniques, such as vascular access, or use of specific stents or closure devices. Although the size of the sheath and guiding catheter were not protocol-mandated in this cohort, the commonly used size was 6-Fr to 8-Fr when a transfemoral approach was used and 6-Fr for transradial interventions. The majority of clinical variables in the JCD were defined according to the National Cardiovascular Data Registry (NCDR), which was sponsored by the American College of Cardiology (ACC) to conduct comparative research in order to determine the factors leading to disparities in PCI management. The NCDR is a large PCI registry system with over 1,000,000 entries for ischemic heart disease and over 500,000 entries for PCI, collected from more than 500 institutions in the US [11].

The study was approved by the institutional review board of Keio University School of Medicine. The patient record was anonymized and de-identified prior to analysis. Major teaching hospitals within the metropolitan Tokyo area were selected for the pilot phase of this study, and the study protocol was approved by the institutional review board at each site. The written consent was obtained by the study participants. Patients were enrolled at the event; all the consecutive PCI procedures during the study period, including failure cases, were registered. Patients aged <18 years were excluded.

The present study was funded by the Kakenhi (Grant-in-Aid for Scientific Research) (No. 21790751). The JCD Steering Committee was responsible for overall study guidance, including the study protocol, data analysis, and interpretation of the results. The Department of Healthcare Quality Assessment of Tokyo University managed the database independently. Keio University School of Medicine Interhospital Cardiology Study Group managed the participating sites and provided a monthly on-site monitoring service to assure data accuracy and completeness throughout the study. During the planning, implementation and reporting of this study, there were no issues such as conflict of interest, conflict of responsibility, or intellectual property rights.

### Information disclosure

Before the launch of the JCD, information about the objectives of the present study, its social significance, and an abstract were provided for clinical trial registration with the University Hospital Medical Information Network, which is recognized by the International Committee of Medical Journal Editors as an “acceptable registry” according to a statement issued in September 2004 (UMIN R000004736).

### Definition of lesion complexity risk score and clinical outcomes

A complex lesion was defined as a treated lesion possessing at least one of the following high-risk angiographic lesion characteristics: bifurcation, chronic total occlusion (CTO), Type C, unprotected left main trunk (UPLMT), and thrombus formation. The size of the main or side branch vessel had to be at least 1.5 mm in diameter, as assessed by diagnostic angiogram, and significant stenosis was defined as a reduction of at least 50% in luminal diameter, by visual assessment. Bifurcation lesions were defined as a division of a vessel into at least two branches, with the plaque extending from at least one of the limbs to the branching point. A CTO lesion was defined as a 100% occluded lesion with complete interruption of antegrade flow (TIMI flow grade 0) that had been present for at least 3 months. Type C lesions were as defined by the American Heart Association/American College of Cardiology (AHA/ACC). UPLMT lesions were left main trunk lesions without patent coronary artery bypass grafts in the left anterior descending artery or the left circumflex artery. Thrombotic lesions were defined as the presence of new ST-segment elevation myocardial infarction (STEMI) as clinical presentation, with cardiac biomarkers exceeding the upper limit of normal, according to the individual hospital’s laboratory parameters. The operator determined the presence of these characteristics at the time of the coronary angiography. Patients were stratified both by absolute complex lesion status (yes/no), and by the total number of the five complex lesion criteria that were present (score 0: no complex lesion, score 1: one complex lesion, score 2: two complex lesions, score 3: three complex lesions, score 4: four complex lesions, and score 5: all complex lesions).

The primary outcome was all-cause in-hospital mortality and secondary outcomes included all in-hospital complications. In-hospital complications collected in the data system included in-hospital death from any cause after PCI; the combined cardiac events included in-hospital cardiac death (in-hospital death, heart failure or cardiogenic shock) [12], periprocedural myocardial infarction (defined as an increase in serum creatine kinase to above normal levels, associated with positive isoenzymes, routinely measured for all patients on the day after the PCI procedure), bleeding with a hemoglobin decrease of more than 3.0 g/dL or blood transfusion, contrast nephropathy, and persistent coronary flow reduction (TIMI flow less than grade 3). Contrast nephropathy was defined, according to the established definition in the literature, as an increase in serum Cr level ≥0.5 mg/dL or ≥25% above baseline values at 30 days after administration of contrast media, in the absence of any other identifiable major kidney insult. Major bleeding was defined as: 1) bleeding requiring a blood transfusion; 2) a decrease in the Hb level by ≥3.0 g/dL due to bleeding from any site, including the percutaneous entry site, retroperitoneum, gastrointestinal tract, genitourinary tract, and other/unknown sites; and 3) the need for intervention/surgery at the bleeding site to reverse/stop or correct the bleeding (such as surgical closure/exploration of the arteriotomy site, balloon angioplasty to seal an arterial tear, or endoscopy with cautery of a gastrointestinal bleed). The latter definition is equivalent to Bleeding Academic Research Consortium Type 3 bleeding.

### Statistical analysis

Patients were stratified by the complexity score (0–5) of the target lesion(s), as described above. Descriptive statistics were calculated on the basis of clinical characteristics and treatment information for the registered patients. Statistical analysis was performed using SPSS version 15 (SPSS, Chicago, IL, USA). When continuous variables were assumed to show a normal distribution, the data were expressed as mean ± SD. When normality was not assumed, the data were expressed as median and interquartile range. Categorical data were summarized in terms of frequency and proportion. The 95% confidence intervals of the mean, median, and proportion values were also calculated. The baseline clinical characteristics of patients were compared using chi-square tests for categorical variables and analysis of variance for continuous variables. A two-tailed p-value <0.05 was considered statistically significant. Multivariate logistic regression analysis was performed to assess the association of increments in each lesion complexity score with in-hospital mortality and complications.

## Results

### Patients and baseline characteristics

**Fig 1:**
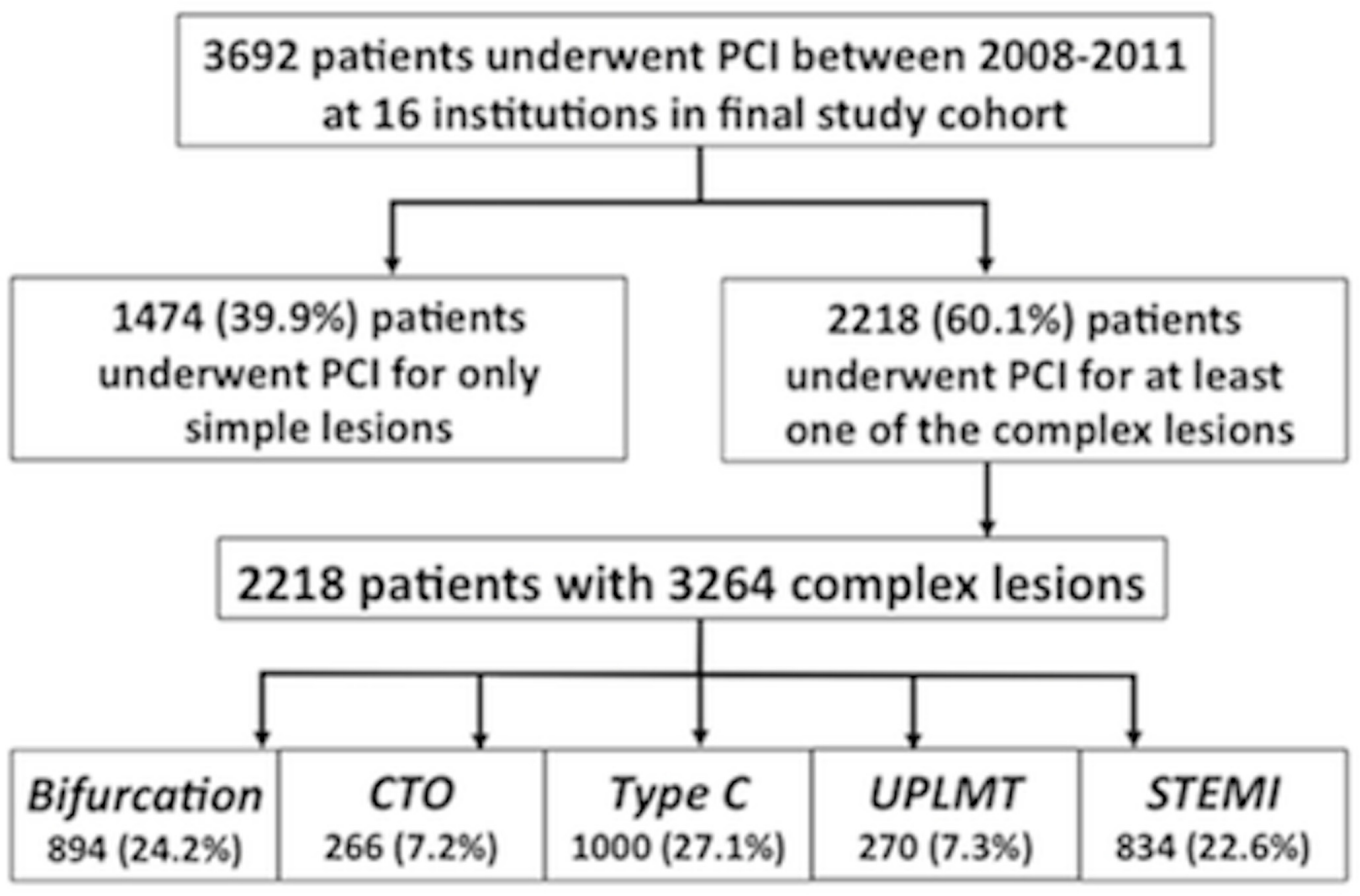
Flowchart showing the patients included in the present analysis. A total of 3692 patients were evaluated.

Among the 3692 patients who underwent PCI procedures between September 1, 2008, and August 31, 2011, and were recorded in the JCD Registry, 2218 (60.1%) underwent revascularization of at least one complex lesion (Fig 1). Of the total of 3264 complex lesions, 894 (24.2%) were bifurcation, 266 (7.2%) were CTO, 1000 (27.1%) were type C, 270 (7.3%) were UPLMT, and 834 (22.6%) were STEMI. Thus, the most common types of complex lesion undergoing revascularization were type C, bifurcation, and STEMI lesions. The distribution of the study patients by complexity score is shown in Fig 1. In total, 1474 patients (39.9%) had no complex lesion attempted (score 0 group), 1375 patients (37.2%) had one complex lesion (score 1 group), 650 patients (17.6%) had 2 complex lesions (score 2 group), 183 patients (5.0%) had 3 complex lesions (score 3 group), and 10 patients (0.3%) had 4 complex lesions attempted (score 4 group). No patients had lesions with all types of complexity (score 5 group).

The clinical and angiographic characteristics of all the groups are shown in Table 1. The mean age of the entire cohort was 67.5 ± 10.8 years and 2935 (79.5%) were male. Overall, 1857 (50.3%) of the patients presented with acute coronary syndromes (ACS) and 1557 (42.2%) underwent non-elective procedures. The relationship between lesion complexity score and baseline variables had an inverted U-shape for male sex and a U-shape for age, especially for patients over 70 years old. Compared to patients in the score 0 group, patients in higher score groups had a lower left ventricular ejection fraction and were more likely to have a multi-vessel lesion in the left anterior descending artery. Patients in higher score groups also tended to present with ACS and heart failure, underwent more emergency PCI procedures, and were treated more frequently with a femoral artery approach, with intra-aortic balloon pump support, and with drug-eluting stents (DES).

**Table 1:** Patient Demographics.

	Score 0 (n = 1474)	Score 1(n = 1375)	Score 2 (n = 650)	Score 3 (n = 183)	Score 4 (n = 10)	P value
Demographics						
Age, yrs	68.42±10.16	66.62±11.10	66.58±11.22	69.43±10.32	72.40±10.95	<0.001
50–59 yrs, n (%)	248 (16.8)	291 (21.2)	132 (20.3)	29 (15.8)	0 (0.0)	0.011
60–69 yrs, n (%)	519 (35.2)	480 (34.9)	228 (35.1)	63 (34.4)	2 (20.0)	0.902
70–79 yrs, n (%)	601 (40.8)	452 (32.9)	217 (33.4)	68 (37.2)	5 (50.0)	<0.001
>80 yrs, n (%)	181 (12.3)	162 (11.8)	67 (10.3)	32 (17.5)	2 (20.0)	0.102
Male, n (%)	1136 (77.1)	1098 (79.9)	533 (82.0)	161 (88.0)	7 (70.0)	0.002
Height, (cm)	161.45±8.92	162.46±9.83	162.40±8.77	163.45±8.33	158.38±7.48	0.005
Weight, (kg)	63.51±12.03	64.86±13.75	63.85±12.78	63.59±12.69	60.73±9.13	0.064
Body mass index, (kg/m2)	24.24±3.35	25.38±26.58	24.13±3.75	23.71±3.23	24.86±2.53	0.316
Clinical history						
Obese (BMI >30), n (%)	75 (5.1)	100 (7.3)	40 (6.2)	9 (4.9)	1 (10.0)	0.155
Hypertension, n (%)	1139 (77.3)	958 (69.7)	446 (68.6)	124 (67.8)	7 (70.0)	<0.001
Hyperlipidemia, n (%)	1031 (69.9)	892 (64.9)	429 (66.0)	106 (57.9)	6 (60.0)	0.003
Diabetes, n (%)	626 (42.5)	554 (40.3)	267 (41.1)	67 (36.6)	5 (50.0)	0.5
Insulin-dependent diabetes, n (%)	139 (9.4)	138 (10.0)	63 (9.7)	12 (6.6)	2 (20.0)	0.469
Current smoking, n (%)	427 (29.0)	523 (38.0)	236 (36.3)	57 (31.1)	2 (20.0)	<0.001
Familiy history of CAD, n (%)	32 (2.2)	45 (3.3)	25 (3.8)	4 (2.2)	0 (0.0)	0.184
Use of antianginal agents, n (%)	350 (23.7)	249 (18.1)	130 (20.0)	32 (17.5)	2 (20.0)	0.004
COPD, n (%)	45 (3.1)	34 (2.5)	19 (2.9)	4 (2.2)	0 (0.0)	0.833
Cancer, n (%)	51 (3.5)	49 (3.6)	23 (3.5)	5 (2.7)	0 (0.0)	0.951
Preoperative Creatinine, (mg/dl)	1.22±1.63	1.18±1.53	1.24±1.72	1.12±1.21	1.03±0.24	0.879
GFR, (ml/min)	87.13±33.03	87.79±31.13	90.13±34.26	89.94±46.56	71.08±15.09	0.211
Hemodialysis, n (%)	57 (3.9)	44 (3.2)	26 (4.0)	4 (2.2)	1 (10.0)	0.48
LVEF, (%)	59.35±12.75	55.81±13.99	54.78±13.18	54.81±13.09	46.86±13.43	<0.001
Cerebro-vascular disease, n (%)	125 (8.5)	98 (7.1)	52 (8.0)	18 89.8)	1 (10.0)	0.595
Peripheral artery disease, n (%)	102 (6.9)	99 (7.2)	49 (7.5)	15 (8.2)	3 (30.0)	0.083
Prior MI, n (%)	417 (28.3)	299 (21.7)	153 (23.5)	37 (20.2)	4 (40.0)	<0.001
Prior PCI, n (%)	651 (44.2)	416 (30.3)	189 (29.1)	62 (33.9)	3 (30.0)	<0.001
Prior CABG, n (%)	98 (6.6)	68 (4.9)	32 (4.9)	7 (3.8)	0 (0.0)	0.167
Prior HF, n (%)	142 (9.6)	81 (5.9)	39 (6.0)	14 (7.7)	2 (20.0)	0.001
Admission presentation						
STEMI, n (%)	0 (0)	508 (36.9)	250 (38.5)	70 (38.3)	6 (60.0)	<0.001
non-STEMI, n (%)	150 (10.2)	82 (6.0)	45 (6.9)	9 (4.9)	1 (10.0)	<0.001
Unstable angina, n (%)	404 (27.4)	221 (16.1)	82 (12.6)	28 (15.3)	1 (10.0)	<0.001
CCS 3, n (%)	378 (25.6)	259 (18.8)	111 (17.1)	29 (15.8)	2 (20.0)	<0.001
CCS 4, n (%)	139 (9.4)	136 (9.9)	59 (9.1)	13 (7.1)	2 (20.0)	0.575
Stable angina, n (%)	501 (34.0)	314 (22.8)	135 (20.8)	38 (20.8)	1 (10.0)	<0.001
Silent ischemia, n (%)	375 (25.4)	223 (16.2)	127 (19.5)	35 (19.1)	1 (10.0)	<0.001
Heart Failure, n (%)	143 (9.7)	125 (9.1)	78 (12.0)	22 (12.0)	3 (30.0)	0.049
NYHA 3, n (%)	76 (5.2)	75 (5.5)	43 (6.6)	12 (6.6)	2 (20.0)	0.194
NYHA 4, n (%)	38 (2.6)	54 (3.9)	33 (5.1)	7 (3.8)	2 (20.0)	0.012
In-hospital presentation						
Staged PCI, n (%)	109 (7.4)	106 (7.7)	46 (7.1)	19 (10.4)	0 (0.0)	0.523
Silent ischemia, n (%)	251 (17.0)	152 (11.1)	101 (15.5)	26 (14.2)	1 (10.0)	<0.001
2 vessel disease, n (%)	623 (42.3)	593 (43.1)	308 (47.4)	106 (57.9)	8 (80.0)	<0.001
3 vessel disease, n (%)	324 (22.0)	339 (24.7)	174 (26.8)	58 (31.7)	4 (40.0)	0.01
LAD, n (%)	432 (29.3)	509 (37.0)	275 (42.3)	93 (50.8)	5 (50.0)	<0.001
Procedure						
Urgent PCI, n (%)	332 (22.5)	296 (21.5)	141 (21.7)	38 (20.8)	2 (20.0)	0.961
Emergent PCI, n (%)	90 (6.1)	421 (30.6)	200 (30.8)	53 (29.0)	4 (40.0)	<0.001
Drug Eluting Stent, n (%)	995 (67.5)	920 (66.9)	402 (61.8)	105 (57.4)	6 (60.0)	0.011
Bare Metal Stent, n (%)	365 (24.8)	408 (29.7)	138 (21.2)	34 (18.6)	1 (10.0)	<0.001
IABP support, n (%)	11 (0.7)	21 (1.5)	21 (3.2)	6 (3.3)	1 (10.0)	<0.001
IVUS use, n (%)	596 (40.4)	470 (34.2)	190 (29.2)	56 (30.6)	0 (0.0)	<0.001
RA approach, n (%)	433 (29.4)	280 (20.4)	112 (17.2)	41 (22.4)	1 (10.0)	<0.001
FA approach, n (%)	989 (67.1)	1065 (77.5)	514 (79.1)	139 (76.0)	8 (80.0)	<0.001
Fluoro Time, (min)	59.35±12.75	55.81±13.99	54.78±13.18	54.81±13.09	46.86±13.43	<0.001

Data are expressed as mean ± SD.

ACS, acute coronary syndrome; BMI, body mass index; CABG, coronary artery bypass grafting; CCS, canadian cardiovascular society; FA, femoral approach; GFR. glomerular filtration rate; HF, heart failure; IABP, intra aorta balloon pump; IVUS, intra vascular ultra sound; MI, myocardial infarction; NSTEMI, non-ST-segment elevation myocardial infarction; PCI, percutaneous coronary intervention; RA, radial approach; STEMI, ST-segment elevation myocardial infarction; UA, unstable angina.

### Clinical outcomes

**Fig 2:**
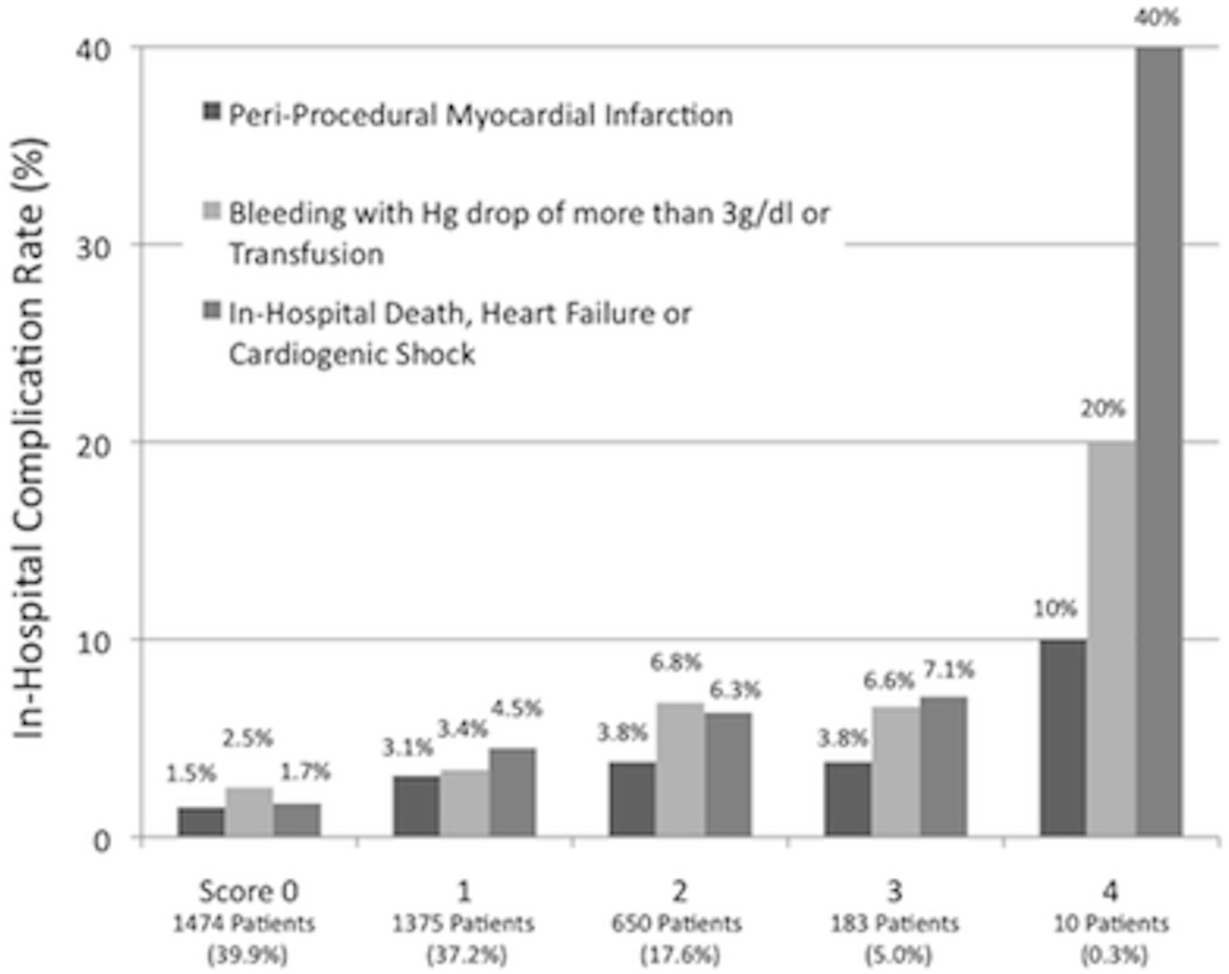
The rates of in-hospital complications.

Details of in-hospital complications are shown in Table 2. Although 3265 (88.4%) procedures were completed successfully, 427 (11.6%) were associated with at least one complication. The rates of in-hospital mortality and complications were higher in the higher score groups compared with the lower score groups. Importantly, patients in higher score groups had significantly higher rates of in-hospital events, including death, heart failure and cardiogenic shock, postoperative myocardial infarction, and bleeding with a hemoglobin decrease of more than 3.0 g/dL or transfusion (Fig 2).

**Table 2:** Periprocedural and In-hospital Complication data.

	Score 0 (n = 1474)	Score 1 (n = 1375)	Score 2 (n = 650)	Score 3 (n = 183)	Score 4 (n = 10)	P value
In hospital mortality, n (%)	11 (0.7)	20 (1.5)	12 (1.8)	2 (1.1)	1 (10.0)	0.02
All complications, n (%)	97 (6.6)	177 (12.9)	118 (18.2)	31 (16.9)	4 (40.0)	<0.001
Cardiogenic shock, n (%)	7 (0.5)	26 (1.9)	19 (2.9)	4 (2.2)	3 (30.0)	<0.001
MI post PCI, n (%)	22 (1.5)	42 (3.1)	25 (3.8)	7 (3.8)	1 (10.0)	0.004
Death/HF/CS, n (%)	25 (1.7)	62 (4.5)	41 (6.3)	13 (7.1)	4 (40.0)	<0.001
Bleeding complications, n (%)	31 (3.1)	117 (11.0)	69 (13.1)	16 (10.3)	2 (28.6)	<0.001
Transfusion, n (%)	24 (1.6)	23 (1.7)	27 (4.2)	7 (3.8)	0 (0)	0.001
Contrast Nephropathy	104 (7.1)	151 (11.0)	98 (15.1)	33 (18.0)	4 (40.0)	<0.001
Introduction of new hemodialysis, n (%)	7 (0.5)	10 (0.7)	5 (0.8)	2 (1.1)	1 (10.0)	0.006
TIMI flow under grade 3, n (%)	30 (2.0)	62 (4.5)	48 (7.4)	13 (7.1)	2 (20.0)	<0.001
Major dissection, n (%)	10 (0.7)	27 (2.0)	16 (2.5)	3 (1.6)	0 (0)	0.012
Coronary perforation, n (%)	7 (0.5)	16 (1.2)	12 (1.8)	3 (1.6)	0 (0)	0.045

CS, Cardiogenic shock; HF, heart failure; MI, myocardial infarction; PCI, percutaneous coronary intervention; TIMI, Thrombolysis In Myocardial Infarction.

The presence of these variables predicted the in-hospital outcome after adjustment for known clinical predictors (Tables 3 and 4; results of the univariate analyses is available in S1 and S2 Tables). Importantly, the in-hospital mortality increased by 1.38 per unit increment in complexity score (odds ratio, OR 1.38; p < 0.001). Furthermore, the risk of an in-hospital complication increased by 1.73 per unit increment in complexity score (OR 1.73; p < 0.001). Of note, there is a partial overlap in the Type C and CTO lesions, and we performed a secondary analysis excluding CTO from our scoring system (S3 and S4 Tables); however, this did not alter our main results and complexity score remained an independent predictor for in-hospital death (OR 1.48; p<0.001) and complication (OR 1.90; p<0.001).

**Table 3:** Multivariable predictors for in-hospital mortality.

	Odds Ratio	Lower 95% CI	Upper 95% CI	P value
Complexity Score (increment by unit)	1.38	1.02	1.88	0.039
Female	1.11	0.56	2.2	0.771
Age over 70 yrs	6.73	2.8	16.19	<0.001
CKD	4.87	2.23	10.6	<0.001
DM	0.72	0.38	1.38	0.322
COPD	2.62	0.82	8.36	0.105
Cerebrovascular Disease	1.39	0.61	3.19	0.435
HF (NYHA4)	2.3	1.07	4.93	0.032
Prior PCI	0.44	0.2	0.94	0.034
Prior CABG	3.63	1.55	8.47	0.003
Prior HF	2.3	1.07	4.93	0.032

CABG, coronary artery bypass grafting; CI, confidence interval; CKD, chronic kidney disease; COPD, chronic obstructive pulmonary disease; DM, diabetes mellitus; HF, heart failure; NYHA, New York Heart Association; PCI, percutaneous coronary intervention.

**Table 4:** Multivariable predictors of any complications.

	Odds Ratio	Lower 95% CI	Upper 95% CI	P value
Complexity Score (increment by unit)	1.73	1.45	2.06	<0.001
Female	1.13	0.75	1.71	0.551
Age over 70 yrs	1.69	1.18	2.42	0.004
CKD	1.93	1.05	3.53	0.035
DM	0.87	0.61	1.25	0.457
COPD	1.69	0.73	3.91	0.217
Cerebrovascular Disease	1.21	0.68	2.15	0.515
HF (NYHA4)	3.71	2.17	6.35	<0.001
Prior PCI	0.37	0.24	0.59	<0.001
Prior CABG	2.19	1.19	4.02	0.012
Prior HF	1.83	1.09	3.1	0.023

CABG, coronary artery bypass grafting; CI, confidence interval; CKD, chronic kidney disease; COPD, chronic obstructive pulmonary disease; DM, diabetes mellitus; HF, heart failure; NYHA, New York Heart Association; PCI, percutaneous coronary intervention.

## Discussion

We developed a simple PCI scoring system, based on angiographic lesion complexity, for predicting the risk of in-hospital mortality and complications. We tested the system against data from 3692 patients enrolled in a multicenter Japanese registry between 2008 and 2011. This complexity scoring system correlated well with in-hospital mortality and complication rates: patients with higher scores exhibited higher event rates compared with lower score groups, while the mortality and complication rate increased by 1.38 and 1.74, respectively, per unit rise in complexity score. The results of this study suggest that quantification of these angiographic characteristics could be of assistance in in-hospital risk stratification and that patients with a high complexity score warrant special attention.

During the last decade, there has been remarkable development in novel devices for PCI, such as first or second generation DES, along with their delivery systems. Hence, the contemporary management of coronary artery diseases has become increasingly dependent on PCI, rather than CABG. However, successful PCI of difficult lesions requires advanced techniques, and the learning curve increases steeply along with the need for greater skill and experience on the part of the operator. Therefore, accurate risk assessment is an essential part of the evaluation in patients undergoing complex PCI. In the present study, each variable showed a tendency to predict in-hospital events (S1 and S2 Tables). When each variable was assigned as a factor to construct a single ‘scoring system’, each unit increment of the score was cumulatively associated with risk for in-hospital events. Our basis for the selection of each of the 5 angiographic variables was primarily clinical. Chosen variables had to be readily available and clinically relevant when performing complex PCI. These variables are thought to directly reflect the complexity of angiographic lesions seen in practice, and are frequently discussed at the bedside and/or catheterization lab when performing the procedure. This in contrast to previously established “complexity” scores (e.g., the SYNTAX score) that aimed to predict the long-term results of PCI compared to CABG [8,10]. This concept is close to that of CHADS2 score for atrial fibrillation.

It is worth emphasizing that the in-hospital complication rate increased approximately twofold in the score 1 group, threefold in the score 2 and 3 groups, and more than tenfold in the score 4 group, compared to patients with simple lesions (score 0 group). Clearly, the presence of complex lesions must always be taken into account in any attempt to forecast the probability of final procedural success and safety. It may also be possible to assign operators appropriately, according to their level of skill, based on the difficulty of dealing with these complex lesions, or to consider referring the patient for CABG. Complex lesions with a low complexity score can usually be treated successfully, and would be good candidates for training purposes.

Previously published studies have indicated a similar trend in the clinical predictors for in-hospital mortality and complications in PCI patients, based on multivariate logistic regression analysis [3,4,7,13–17]. For example, old age, female sex, current or past heart failure symptoms, renal failure, and peripheral artery disease were included in all of the studies. It is noteworthy that our angiographic complexity score continued to be a significant predictor of in-hospital events, even after adjusting for known clinical predictors. Therefore, lesion complexity should be recognized as an important risk factor, in addition to variables that are related to the patient’s background.

In this study, the overall in-hospital mortality rate was 1.2%. This mortality rate is relatively high compared with previous studies, which reflects the high percentage (about 40%) of patients with ACS, cardiogenic shock, or cardiopulmonary arrest. A comparison of the results among patients with stable CAD and those with ACS, shock, or cardiopulmonary arrest will be the subject of further analysis.

Several important limitations of the present analysis should be discussed. The first is sample size. The high score groups contained only small numbers of patients and the full score group had none. Additional validation in the high score groups with larger samples might be needed. A second limitation is that we did not analyze the potential relationship between hospital or operator procedure volume and in-hospital complications. In particular, there may be a relationship between an institution’s or operator’s procedure experience and volume and the outcomes of PCI for complex lesions. Third, it may not have been possible to distinguish Type C lesions from bifurcation lesions and CTO.　However, the number of patients with Type C lesions was small, less than 10%. Fourth, we did have clear discrimination between Type C and heavily calcified lesions. In usual practice, defining universal ‘heavy’ calcification nor its quantification would be a major challenge. Because our goal was to generate a bedside-friendly tool to assess complexity of PCI that would predict the clinical outcome, we chose to incorporate type C lesion as a variable in the scoring system, which is widely appreciated among interventional cardiologists. Lastly, the odds ratio for each complex lesion in our scoring system was not evaluated separately using multivariate logistic regression analysis. We believe that the risk weights for each type of complex lesion are likely to be slightly different. Further analysis, multiplication of the predicted risk for each risk score by the odds ratio for the in-hospital mortality and complications could lead to an improved risk score for evaluation in future studies.

### Conclusions

Accurate risk assessment can aid in the identification of patients who are at high risk of an in-hospital event. The proposed complexity score was cumulatively associated with in-hospital mortality and complication rate and could be used for event prediction in PCI patients. PCI operators should take special care in order to perform PCI successfully in these complex lesions.

## Supporting Information

S1 Table Univariable predictors for in-hospital mortality. (DOCX)

S2 Table Univariable predictors of any complications. (DOCX)

S3 Table Multivariable predictors of in-hospital mortality without CTO lesion. (DOCX)

S4 Table Multivariable predictors of any complications without CTO lesion. (DOCX)
