# Supplementary material for: Angiographic Lesion Complexity Score and In-Hospital Outcomes after Percutaneous Coronary Intervention
Source: PLoS One. 2015 Jun 29;10(6):e0127217. doi: 10.1371/journal.pone.0127217 (PMC4487684; doi:10.1371/journal.pone.0127217)
Supplement: S1 Table — (DOCX) [file pone.0127217.s001.docx]

**S1 Table.** Univariable predictors for in-hospital mortality

|  | Odds Ratio | Lower 95% CI | Upper 95% CI | P value |
| --- | --- | --- | --- | --- |
| Bifurcation lesion | 1.19 | 0.58 | 2.41 | 0.638 |
| CTO | 0.45 | 0.09 | 2.21 | 0.326 |
| Type C | 1.56 | 0.78 | 3.09 | 0.206 |
| UPLMT | 0.61 | 0.17 | 2.23 | 0.451 |
| STEMI | 4.50 | 2.26 | 8.93 | <0.001 |

CTO = chronic total occlusion; STEMI = ST-segment elevation myocardial infarction; UPLMT = unprotected left main trunk.
