# Supplementary material for: Angiographic Lesion Complexity Score and In-Hospital Outcomes after Percutaneous Coronary Intervention
Source: PLoS One. 2015 Jun 29;10(6):e0127217. doi: 10.1371/journal.pone.0127217 (PMC4487684; doi:10.1371/journal.pone.0127217)
Supplement: S2 Table — (DOCX) [file pone.0127217.s002.docx]

**S2 Table.** Univariable predictors of any complications

|  | Odds Ratio | Lower 95% CI | Upper 95% CI | P value |
| --- | --- | --- | --- | --- |
| Bifurcation lesion | 1.04 | 0.69 | 1.57 | 0.852 |
| CTO | 0.92 | 0.44 | 1.89 | 0.811 |
| Type C | 2.15 | 1.47 | 3.12 | <0.001 |
| UPLMT | 1.06 | 0.55 | 2.03 | 0.870 |
| STEMI | 6.21 | 4.17 | 9.26 | <0.001 |

CTO = chronic total occlusion; STEMI = ST-segment elevation myocardial infarction; UPLMT = unprotected left main trunk.
