# Supplementary material for: Angiographic Lesion Complexity Score and In-Hospital Outcomes after Percutaneous Coronary Intervention
Source: PLoS One. 2015 Jun 29;10(6):e0127217. doi: 10.1371/journal.pone.0127217 (PMC4487684; doi:10.1371/journal.pone.0127217)
Supplement: S3 Table — (DOCX) [file pone.0127217.s003.docx]

**S3 Table.** Multivariable predictors of in-hospital mortality without CTO lesion

|  | Odds Ratio | Lower 95% CI | Upper 95% CI | P value |
| --- | --- | --- | --- | --- |
| Complexity Score (increment by unit) | 1.48 | 1.05 | 2.08 | <0.001 |
| Female | 0.79 | 0.55 | 2.22 | 0.015 |
| Age over 70 yrs | 6.43 | 2.66 | 15.55 | <0.001 |
| CKD | 5.02 | 2.26 | 11.18 | <0.001 |
| DM | 0.64 | 0.33 | 1.26 | 0.199 |
| COPD | 3.28 | 1.07 | 10.02 | 0.037 |
| Cerebrovascular Disease | 1.69 | 0.74 | 3.86 | 0.215 |
| HF (NYHA4) | 3.66 | 1.49 | 9.00 | 0.005 |
| Prior PCI | 0.46 | 0.21 | 1.00 | 0.049 |
| Prior CABG | 3.84 | 1.60 | 9.17 | 0.003 |
| Prior HF | 2.59 | 1.21 | 5.57 | 0.015 |

CABG = coronary artery bypass grafting; CI = confidence interval; CKD = chronic kidney disease; COPD = chronic obstructive pulmonary disease; DM = diabetes mellitus; HF = heart failure; NYHA = New York Heart Association; PCI = percutaneous coronary intervention.
