# Supplementary material for: Angiographic Lesion Complexity Score and In-Hospital Outcomes after Percutaneous Coronary Intervention
Source: PLoS One. 2015 Jun 29;10(6):e0127217. doi: 10.1371/journal.pone.0127217 (PMC4487684; doi:10.1371/journal.pone.0127217)
Supplement: S4 Table — (DOCX) [file pone.0127217.s004.docx]

**S4 Table.** Multivariable predictors of any complications without CTO lesion

|  | Odds Ratio | Lower 95% CI | Upper 95% CI | P value |
| --- | --- | --- | --- | --- |
| Complexity Score (increment by unit) | 1.90 | 1.56 | 2.30 | <0.001 |
| Female | 1.12 | 0.73 | 1.71 | 0.602 |
| Age over 70 yrs | 1.61 | 1.11 | 2.34 | 0.012 |
| CKD | 1.98 | 1.06 | 3.71 | 0.032 |
| DM | 0.88 | 0.61 | 1.29 | 0.518 |
| COPD | 1.88 | 0.82 | 4.32 | 0.136 |
| Cerebrovascular Disease | 1.42 | 0.79 | 2.56 | 0.243 |
| HF (NYHA4) | 3.82 | 2.18 | 6.67 | <0.001 |
| Prior PCI | 0.43 | 0.27 | 0.67 | <0.001 |
| Prior CABG | 1.82 | 0.92 | 3.59 | 0.083 |
| Prior HF | 2.00 | 1.16 | 3.42 | 0.012 |

CABG = coronary artery bypass grafting; CI = confidence interval; CKD = chronic kidney disease; COPD = chronic obstructive pulmonary disease; DM = diabetes mellitus; HF = heart failure; NYHA = New York Heart Association; PCI = percutaneous coronary intervention.
